# Enhancing the Physical Properties of Calcium Silicate Cement Modified with Elastin-like Polypeptides and Bioactive Glass

**DOI:** 10.3390/jfb16050188

**Published:** 2025-05-19

**Authors:** Jiyoung Kwon, Hyun-Jung Kim

**Affiliations:** 1 Department of Conservative Dentistry, Kyung Hee University Dental Hospital, Seoul 02453, Republic of Korea; jykt55@naver.com; 2 Department of Conservative Dentistry, School of Dentistry, Kyung Hee University, Seoul 02453, Republic of Korea

**Keywords:** bioactive glass, calcium silicate cements, elastin-like polypeptides

## Abstract

Conventional calcium silicate cement (CSC) formulations often exhibit insufficient mechanical strength and low initial stability. This study aimed to develop an organic–inorganic hybrid biomaterial by incorporating an elastin-like polypeptide (ELP) (V125E8) and bioactive glass (BG) (63S) into CSC to improve its mechanical properties and wash-out resistance during the initial setting. Experimental groups included ProRoot MTA (Dentsply Sirona, USA) as a control (0BG), inorganic hybrids containing BG (2% or 5%; 2BG, 5BG), and organic–inorganic hybrids combining BG (2% or 5%; 2BG-L, 5BG-L) with a 10 wt% ELP solution. The compressive strength, microhardness, and wash-out resistance of the specimens were evaluated. The organic–inorganic hybrid groups (2BG-L and 5BG-L) exhibited significantly higher compressive strength and microhardness than the control (0BG) and inorganic-only groups (2BG and 5BG). Additionally, the incorporation of ELP markedly improved wash-out resistance, minimizing material disintegration during the initial setting in aqueous environments. The organic–inorganic hybrid groups (2BG-L and 5BG-L) exhibited significantly higher compressive strength and microhardness than the control (0BG) and inorganic-only groups (2BG and 5BG). Additionally, the incorporation of ELP markedly improved wash-out resistance, minimizing material disintegration during the initial setting in aqueous environments.

## 1. Introduction

Calcium silicate cements (CSCs) are important biomaterials in contemporary endodontics, owing to their exceptional biocompatibility, superior sealing capabilities, and capacity to stimulate hard tissue formation [1]. Specifically, ProRoot MTA, which is the most widely studied and clinically used CSC in dentistry, consists primarily of tricalcium silicate (Ca_3_SiO_5_, alite), dicalcium silicate (Ca_2_SiO_4_, belite), tricalcium aluminate (Ca_3_Al_2_O_6_), bismuth oxide (Bi_2_O_3_, for radiopacity), and calcium sulfate (CaSO_4_, as a setting regulator) [2]. Upon hydration, these phases react with water to form a calcium silicate hydrate (C-S-H) gel and calcium hydroxide, which are responsible for the material’s mechanical strength and bioactivity. The microstructure of set MTA is characterized by an interlocking network of C-S-H gel with embedded unreacted particles and crystalline phases, as described in previous studies [2,3,4].

Despite these favorable biological characteristics, conventional CSCs possess several inherent limitations, including prolonged setting times, susceptibility to washing out during the initial setting period, and insufficient mechanical properties, such as relatively low compressive strength and microhardness [5,6,7]. These drawbacks significantly limit their clinical efficacy, particularly in scenarios that require rapid setting, enhanced mechanical integrity, and improved handling properties.

To address these limitations, considerable research efforts have been directed toward the development of hybrid biomaterials incorporating organic and inorganic components [8,9]. Organic–inorganic hybrid materials represent an innovative class of biomaterials that synergistically combine the advantages of their constituent phases [10,11,12,13]. Specifically, the incorporation of organic polymers can enhance handling characteristics, flexibility, and biological interactions at cellular interfaces [14,15], whereas inorganic additives can significantly improve mechanical strength, bioactivity, and osteogenic potential [10,16].

In this context, elastin-like polypeptides (ELPs) have garnered substantial attention as organic additives, due to their inherent biocompatibility, biodegradability, thermoresponsive behavior, and ability to modulate cellular responses [17,18]. Elastin is a naturally occurring protein-based polymer that is widely distributed in connective tissues, such as the skin, heart, bladder, blood vessels, cartilage, etc. [19]. ELPs are repetitive artificial polypeptides that mimic the properties of natural elastin [20]. Among various ELP variants, V125E8—comprising 125 repeating pentapeptide sequences (Val-Pro-Gly-Val-Gly) with octaglutamic acid residues at the C-terminus—was selected for this study [17]. This ELP variant exhibits a physiologically relevant transition temperature (between 31 and 33 °C) [17], making it suitable for clinical application as an injectable or moldable biomaterial. Previous studies have demonstrated that V125E8 additions significantly enhance the mechanical performance and bonding characteristics of composite biomaterials [21,22,23]. As such, its incorporation into CSC is anticipated to improve the workability of the material during clinical manipulation and increase its structural integrity after setting.

Concurrently, bioactive glass (BG), particularly the 63S variant composed primarily of silica (SiO_2_), calcium oxide (CaO), and phosphorus pentoxide (P_2_O_5_), has been extensively investigated as an inorganic additive, due to its remarkable bioactivity and osteoconductivity [24,25]. BG facilitates rapid ion exchange with physiological fluids, leading to apatite formation on material surfaces—a critical factor for bone regeneration and mineralization [26,27,28]. Furthermore, previous studies have indicated that adding BG to CSCs enhances the compressive strength and push-out bond strength to root dentin, while simultaneously promoting biological interactions conducive to tissue regeneration [29,30].

Extensive research has been conducted on the individual applications of ELP and BG in biomaterials. ELPs have demonstrated remarkable versatility in tissue engineering applications, due to their biocompatibility, biodegradability, and tunable mechanical properties [17,19]. Similarly, BG has been widely studied for its ability to promote bone regeneration and mineralization [20]. Hench and colleagues demonstrated that 45S5 Bioglass^®^ could form strong interfacial bonds with both hard and soft tissues, stimulating osteogenesis [31]. More recently, El-Fiqi A. et al. found that incorporating BG nanoparticles into calcium phosphate cements significantly improved their mechanical properties and bioactivity [32]. These studies highlight the potential of ELP and BG as individual components in biomaterials. However, research on their synergistic effects when combined in a single-material system, particularly for endodontic applications, remains limited. This gap in knowledge provides a compelling rationale for our current investigation into the development of an organic–inorganic hybrid biomaterial incorporating both ELP and BG.

Considering the advantages of ELP and BG additions individually, we hypothesized that their combined incorporation into CSC would yield an organic–inorganic hybrid biomaterial with superior physical properties to conventional CSC formulations. Systematic evaluations of the synergistic effects of simultaneous ELP and BG additions on the physical characteristics of endodontic materials are scarce. Therefore, the primary objective of this study was to investigate how the additions of V125E8 ELP as an organic component and 63S BG as an inorganic component influences the physical properties—specifically compressive strength, microhardness, and wash-out resistance—of hybridized CSC formulations. We aimed to determine whether this organic–inorganic hybrid material can effectively overcome the limitations associated with traditional CSC materials by enhancing their mechanical integrity and stability under physiological conditions.

The null hypothesis for this study is as follows:

The incorporation of ELP (V125E8) and BG (63S) into CSC does not significantly improve its mechanical properties or wash-out resistance compared to conventional CSC.

## 2. Materials and Methods

### 2.1. Materials

Composite materials were produced from three main components. The core CSC material was ProRoot MTA (Dentsply Sirona, Tulsa, OK, USA), which is commonly used to prepare endodontic fillings. When ProRoot MTA interacts with water, the primary reaction involves tricalcium silicate, which undergoes hydration according to the following equation:

2(3CaO·SiO_2_) + 7H_2_O → 3CaO·2SiO_2_·4H_2_O + 3Ca(OH)_2_ + heat

This reaction produces calcium silicate hydrate (C-S-H) gel, which is the primary binding component, and calcium hydroxide, which contributes to the material’s bioactivity through the release of calcium ions and the creation of an alkaline environment [2,33].

The inorganic component was 63S BG (Bonding Chemical, Katy, TX, USA). It was added to the core CSC material at concentrations of 0, 2, and 5 wt% to prepare samples 0BG, 2BG, and 5BG, respectively. Each powder was mixed with deionized water at a liquid-to-powder (L/P) ratio of 0.3 [34,35]. The organic component was V125E8 ELP, which was synthesized following established protocols. A 10 wt% ELP solution was incorporated into the CSC and CSC/BG powders at an L/P ratio of 0.3 to prepare samples 0BG-L, 2BG-L, and 5BG-L, respectively. Thus, the L/P ratio was constant for all samples. The experimental groups are listed in Table 1. The detailed chemical compositions and proportions of all materials used in each experimental group—including ProRoot MTA, BG (63S), and ELP (V125E8)—are summarized in Table 2.

### 2.2. Synthesis of V125E8

The elastin-like polypeptide (ELP) V125E8 was synthesized using established genetic engineering techniques [17,18]. V125E8 consists of 125 repetitions of the pentapeptide sequence Val-Pro-Gly-Val-Gly (VPGVG), with eight glutamic acid (E) residues added as a functional group at the C-terminus. This specific design imparts unique thermoresponsive properties to the polypeptide. The synthesis process began with the creation of the DNA sequence encoding V125E8. This was accomplished through the annealing and ligation of synthetic oligonucleotides (IDT, Inc., Coralville, IA, USA). The resulting DNA construct was then inserted into a modified pET28b expression vector (EMD Millipore, Gibbstown, NJ, USA), which contained specific N-terminal and C-terminal DNA sequences to facilitate expression and purification. The recombinant plasmid was subsequently transformed into BLR (DE3) *E. coli* (EMD) for protein expression. The transformed *E. coli* specimens were cultured in Terrific Broth medium at 37 °C for 24 h to allow for ELP production. Following cultivation, the bacteria were harvested by centrifugation and resuspended in a buffer containing 10 mM Tris-Cl and 2 mM EDTA (pH 8.0).

Cell lysis was performed using probe sonication, applying three 30 s pulses with 2 min intervals between each sonication (Sonicator 3000, Misonix, Inc., Farmingdale, NY, USA). After lysis, cellular debris was removed by centrifugation, and the supernatant was treated with polyethyleneimine (Sigma-Aldrich, Steinheim, Germany) to precipitate contaminating DNA. The ELP was then purified using three rounds of inverse transition cycling, a method described by Meyer and Chilkoti [18]. This technique exploits the temperature-dependent phase transition behavior of ELPs, allowing for efficient separation from other cellular proteins. The purified V125E8 was dialyzed against deionized water to remove any remaining salts, and then lyophilized to obtain the final product in powder form. The synthesized V125E8 exhibits a transition temperature between 31 and 33 °C, making it suitable for clinical applications. For the preparation of hybrid CSC, a 10 wt% solution of V125E8 in deionized water was used as the liquid phase. This concentration was chosen based on previous studies demonstrating optimal handling and mechanical properties for the resulting composites [21,34].

### 2.3. Compressive Strength Test

To examine the compressive strength, 30 specimens were prepared for each group in custom-made plastic molds (diameter (D) = 3 mm, height (H) = 7 mm) with a Teflon (The Chemours Company, Wilmington, DE, USA) coating. The molds were stored for 2 d in 100% humidity chambers at 37 °C. Subsequently, the specimens were removed from the molds and stored in simulated body fluid (SBF) for 14 d, with the solution replaced every 2 d. The compressive strengths of the specimens were measured using a universal testing machine (AGS-X; Shimadzu, Kyoto, Japan) at a crosshead speed of 1 mm/min. The maximum load (N) required to fracture each sample was measured, and the compressive strength was calculated in MPa.

### 2.4. Microhardness

Five cylindrical specimens (D = 10 mm, H = 1.5 mm) were prepared for each group. A load of 490 mN was applied to the specimens for 10 s using the pyramidal diamond indenter of a microhardness tester (HMV-2; Shimadzu). The representative indentation images for microhardness measurements were captured using the built-in CCD camera of the microhardness tester (HMV-2). After pyramidal diamond indenter application, digital images were automatically captured at 40× magnification. The images were then processed using Shimadzu HMV-G series integrated image analysis software to measure the diagonal lengths of the indentations accurately. This digital imaging system allows for precise measurement of the indentation dimensions, which are critical for calculating the Vickers hardness number (VHN). The VHN was obtained using the following equation: VHN = 1854.4 P/d^2^, where P is the applied load in grams and d is the average length of the indentation in millimeters. The VHN was measured at five points on each specimen, with each measurement point at a constant distance from the others, resulting in a total of 25 measurements per group.

### 2.5. Wash-Out Resistance

Specimens (D = 20 mm, H = 1.5 mm) were prepared using molds, followed by storage for 2 d in 100% humidity chambers at 37 °C. Subsequently, the specimens were removed from the molds and stored in SBF for 14 d, with the solution replaced every 2 d. A solution of 4-(2-hydroxyethyl)-1-piperazineethanesulfonic acid (HEPES; Sigma Aldrich, St. Louis, MO, USA) was used to assess the wash-out resistance of the samples at various time intervals (5 min, 1 h, and 24 h). The specimens were dried for 24 h, and the remaining material was weighed to calculate the solubility. This test was repeated three times for each group.

### 2.6. Statistical Analysis

Data were analyzed using one-way analysis of variance (ANOVA), and Tukey’s test was used for post hoc comparisons. The level of significance was set at α = 0.05. All statistical analyses were performed using GraphPad Prism 10.2.3 (GraphPad Software Inc., San Diego, CA, USA).

## 3. Results

### 3.1. Compressive Strength

**Figure 1:**
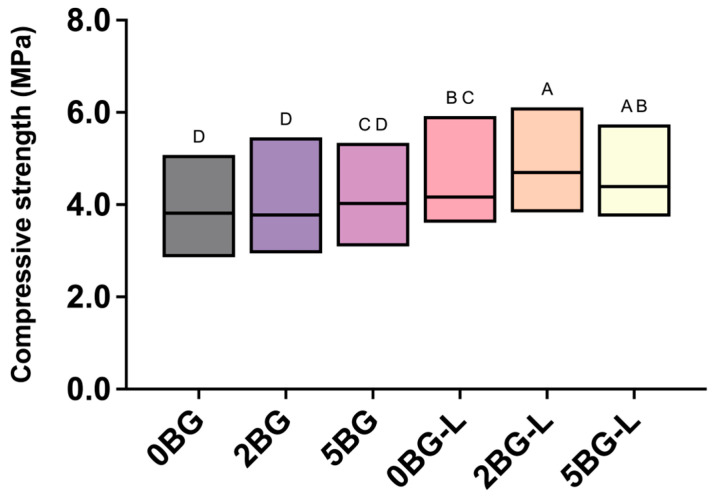
Compressive strengths of experimental calcium silicate cements (CSCs). The same uppercase implies no significant difference (*p* > 0.05).

The results of the compressive strength tests are shown in Figure 1. Group 2BG-L showed a significantly higher compressive strength than the other experimental groups (*p* ≤ 0.0069), except for group 5BG-L (*p* = 0.4011). The compressive strengths of the CSC/BG groups (2BG and 5BG) were not significantly different to that of the positive control (0BG) (*p* ≥ 0.8081).

### 3.2. Microhardness

**Figure 2:**
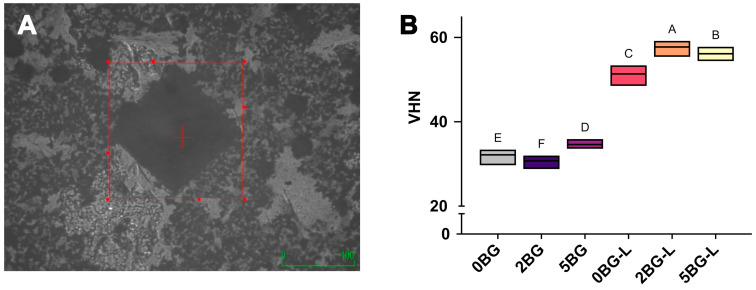
(**A**) Representative image of microhardness measurement. (**B**) Vickers hardness numbers (VHN) of experimental calcium silicate cements (CSCs). The different uppercase implies statistically significant difference (*p* ≤ 0.05).

A representative image of the indentation used for microhardness measurements is provided in Figure 2A. As shown in Figure 2B, group 2BG-L had the highest VHN among all groups (*p* ≤ 0.0058).

### 3.3. Wash-Out Resistance

**Figure 3:**
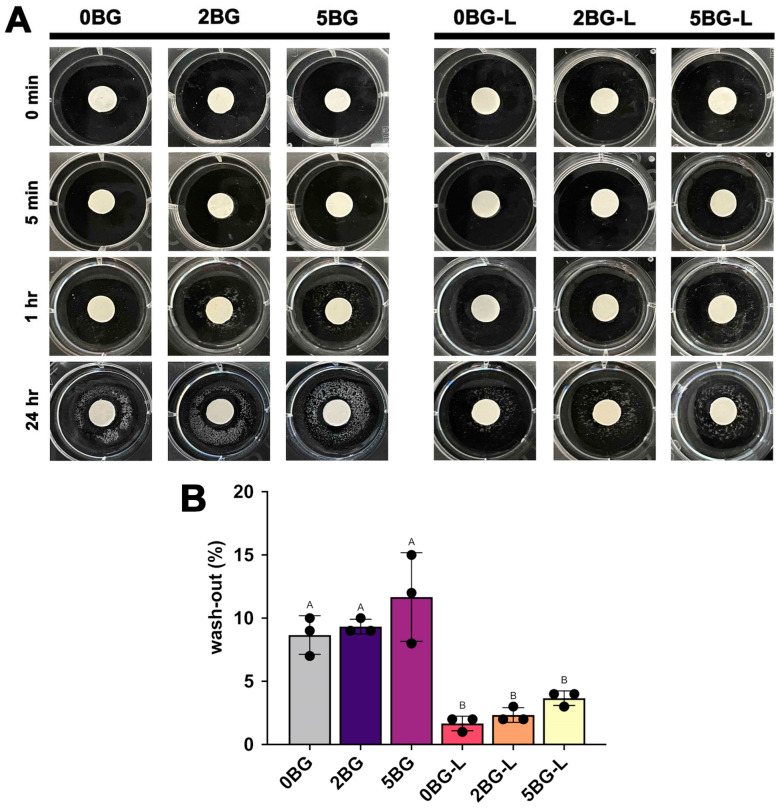
(**A**) Representative images of wash-out test. (**B**) Wash-out proportions of experimental calcium silicate cements (CSCs). The same uppercase implies no significant difference (*p* > 0.05).

The results of the wash-out resistance tests are shown in Figure 3A,B. Groups 0BG, 2BG, and 5BG exhibited increasing disintegration with an increase in immersion time in HEPES solution. By contrast, the ELP-supplemented groups (0BG-L, 2BG-L, and 5BG-L) showed less material disintegration (*p* ≤ 0.0259).

## 4. Discussion

The present study aimed to develop an organic–inorganic hybrid biomaterial by incorporating ELP (V125E8) as an organic additive and 63S BG as an inorganic additive into CSC. The rationale behind this approach was to overcome the inherent limitations of conventional CSCs, such as inadequate mechanical properties, challenging handling characteristics, and susceptibility to washing out during the initial setting process. Our findings demonstrate that the simultaneous additions of ELP and BG significantly enhanced the compressive strength, microhardness, and wash-out resistance of CSC. Based on the results of this study, the null hypothesis was rejected. The incorporation of ELP (V125E8) and BG (63S) into CSC significantly improved both its mechanical properties—including compressive strength and microhardness—and its wash-out resistance compared to the control group.

Regarding the compressive strength, groups containing both ELP and BG (2BG-L and 5BG-L) exhibited significantly higher values than the control (0BG) and inorganic-only groups (2BG and 5BG). Specifically, the median compressive strengths (MPa) and interquartile ranges (IQR, 25th–75th percentile) were as follows: 0BG, 26.97 (23.34–30.62); 2BG, 26.70 (24.51–28.73); 5BG, 28.44 (26.70–29.35); 2BG-L, 33.21 (31.22–35.91); and 5BG-L, 31.04 (29.27–33.91). These data, summarized from the box plot, demonstrate the superior performance of the organic–inorganic hybrid groups. This improvement might be attributed to the synergistic effects of the organic polymer network provided by ELP and the reinforcing action of BG. Previous studies have reported that BG incorporation enhances the mechanical performance of CSC by densifying the cement matrix and promoting apatite formation [30,36]. Furthermore, ELP (V125E8), characterized by its thermoresponsive behavior and ability to form stable polymeric networks at physiological temperatures (31–33 °C), likely contributes to internal structural stability and improved mechanical integrity [22,23]. The transformation behavior of ELP, shifting from a transparent solution below its critical temperature to an opaque aggregate above it, likely plays a crucial role in binding the CSC matrix and structurally reinforcing it, thereby contributing to the enhanced mechanical properties observed in the hybrid materials [17,21,22,37]. Thus, our results align well with the existing literature, while uniquely demonstrating the additional benefit derived from combining organic and inorganic components within the CSC matrix [13,35,38].

The microhardness results further confirm the beneficial effects of hybridization. The organic–inorganic hybrid group 2BG-L showed a significantly higher VHN than the other groups. This improvement likely arises from the homogeneous polymeric network formed by ELP during hydration, which enhances cohesion within the cement matrix. Moreover, BG particles release bioactive ions that facilitate surface apatite formation, further increasing the surface hardness [39]. These findings are consistent with previous reports highlighting improved surface properties upon BG incorporation [36,39]; moreover, our study demonstrates that adding an organic polymeric component, such as ELP, can further amplify these beneficial effects.

The results of our study demonstrate a nuanced relationship between the composition of organic–inorganic hybrid CSCs and their physical properties. While the compressive strength tests showed a general trend of enhanced performance in the organic–inorganic hybrid groups compared to those with only inorganic additives, the differences were not significantly affected by varying concentrations of BG. However, the microhardness results revealed a more refined pattern, with the 2 wt% BG-containing hybrid group (2BG-L) exhibiting the highest VHN. This finding suggests that there exists an optimal ratio for BG incorporation that maximizes the synergistic effects between ELP and BG within the CSC matrix. The discrepancy between the compressive strength and microhardness results highlights the complexity of these hybrid systems and emphasizes the importance of considering multiple physical properties when optimizing biomaterial compositions. By identifying this optimal concentration for microhardness, this study provides valuable insights for fine-tuning the formulation of these hybrid cements to achieve superior overall performance in clinical applications.

Notably, our investigation revealed that the incorporation of ELP markedly enhances wash-out resistance. In our wash-out tests using HEPES solution at various immersion intervals (5 min, 1 h, and 24 h), conventional CSC (0BG) and inorganic-only groups (2BG and 5BG) exhibited significant disintegration over time. In contrast, all groups containing ELP (0BG-L, 2BG-L, and 5BG-L) demonstrated remarkable stability with minimal disintegration. These results clearly indicate that ELP provides early structural integrity during the initial setting period. The mechanism underlying this improvement can be explained by the thermoresponsive nature of V125E8; at physiological temperatures (36–37 °C), ELP undergoes rapid gelation and forms a cohesive polymeric network within the cement matrix [17]. This polymeric network effectively stabilizes particles during hydration reactions and significantly reduces susceptibility to washing out under aqueous conditions. Given that wash-out susceptibility is a critical clinical limitation of conventional CSC, particularly in scenarios involving moisture or bleeding, our findings suggest that incorporating ELP could substantially enhance clinical performance by ensuring material stability during the critical early setting phase.

It is worth noting that, although BG incorporation generally enhances the mechanical properties and bioactivity of CSC, excessive addition of BG can potentially compromise these physical properties. Previous studies have shown that high concentrations of BG can lead to decreased mechanical strength, owing to the formation of loose attachments between the BG particles and the cement matrix [30,40]. This highlights the importance of optimizing the BG concentration in hybrid materials to achieve a balance between enhanced bioactivity and mechanical integrity. In our study, concentrations of 2 and 5 wt% BG were selected, based on previous research that indicated that these levels can effectively improve mechanical properties without significantly compromising them [30,38].

Clinically, these improvements in mechanical strength, hardness, and wash-out resistance hold considerable significance for endodontic applications, such as root-end filling materials in apical surgery, or perforation repair materials, particularly in situations where bleeding control is challenging [41,42]. The enhanced early-stage stability provided by ELP may greatly facilitate handling in moist clinical environments and reduce procedural complications associated with material disintegration.

Despite these promising outcomes, our study has several limitations that warrant further investigation. Notably, this study primarily focused on evaluating the physical properties of the hybrid biomaterials, such as compressive strength, microhardness, and wash-out resistance. The biological effects of these materials, including cytotoxicity, cell proliferation, and osteogenic differentiation, have been explored in a separate study [25]. Furthermore, although the setting time of CSCs is a critical factor influencing their clinical applicability, this study did not include experimental data on setting time. Our primary focus was on early-stage mechanical integrity and wash-out resistance. Previous studies have reported that the relatively long setting time of commercial materials such as ProRoot MTA can limit their widespread clinical use [38,43]. We acknowledge this as a limitation of the present work and plan to include comprehensive setting time analyses in future studies to provide a more complete characterization of these hybrid materials. It is important to note that, while phase composition and microstructural analyses (e.g., XRD, SEM) could provide deeper insights into the material’s crystalline structure and morphology, this study focused on evaluating macro-scale physical properties that directly impact clinical handling and performance. Future studies, incorporating comprehensive microstructural characterization, would further elucidate the underlying mechanisms of property enhancement observed in this work.

Our research complements these findings by providing a comprehensive understanding of the physical characteristics that are crucial for clinical performance. Additionally, long-term stability tests under simulated physiological conditions should be conducted to evaluate durability over extended periods. Future research involving animal models or clinical trials would also be valuable to validate these laboratory findings under clinical conditions.

## 5. Conclusions

This study demonstrates that incorporating an ELP (V125E8), together with BG (63S), into CSC significantly improves its compressive strength, microhardness, and wash-out resistance. This enhancement is attributed to the synergistic effects of ELP’s thermoresponsive polymeric network formation, which provides early structural integrity, as well as the ability of BG to promote apatite formation, thereby enhancing mechanical properties and bioactivity. These findings provide foundational evidence for the further development of advanced hybrid biomaterials for enhanced endodontic clinical applications.

## Figures and Tables

**Table 1:** Preparation of experimental groups.

	Groups	Liquid	Powder
Positive control	0BG	DW	100% MTA
Inorganic hybrid CSC	2BG	DW	98% MTA + 2% BG
	5BG	DW	95% MTA + 5% BG
Organic–inorganic hybrid CSC	0BG-L	ELP	100% MTA
	2BG-L	ELP	98% MTA + 2% BG
	5BG-L	ELP	95% MTA + 5% BG

BG, bioactive glass; CSC, calcium silicate cement; DW, deionized water; ELP, 10 wt% elastin-like polypeptide solution; MTA, mineral trioxide aggregate; %, wt%.

**Table 2:** Component overview of hybrid cement materials.

Component	Description	Composition/Details
Base Material	ProRoot MTA (Dentsply Sirona, Tulsa, OK, USA)	-75% White Portland cement (Ca_3_SiO_5_, Ca_2_SiO_4_, Ca_3_Al_2_O_6_) -20% Bi_2_O_3_ -5% CaSO_4_
Inorganic Additive	63S Bioactive Glass (Bonding Chemical, Katy, TX, USA)	-63% SiO_2_ -28% CaO -9% P_2_O_5_ -Particle size < 40 μm, purity 99.9%
Organic Additive	Elastin-like Polypeptide (V125E8)	-10 wt% aqueous solution -Synthesized via recombinant protein expression

## Data Availability

The original contributions presented in the study are included in the article, further inquiries can be directed to the corresponding author.
